# Difference of glucose and lipid metabolism abnormalities and body fat between the Chinese and USA teenagers

**DOI:** 10.7189/jogh.13.04041

**Published:** 2023-05-19

**Authors:** Yiwen Huang, Liwang Gao, Hong Cheng, Xi Wang, Hongbo Dong, Yinkun Yan, Xiaoyuan Zhao, Junting Liu, Xinying Shan, Jie Mi

**Affiliations:** 1 Center for Non-communicable Disease Management, Beijing Children's Hospital, Capital Medical University, National Center for Children's Health, Beijing, China; 2 Department of Epidemiology, Capital Institute of Pediatrics, Beijing, China

## Abstract

**Background:**

Comparing body fat and the effect of body fat on metabolic abnormalities in Chinese and USA teenagers may provide clues for the early prevention and control of cardiovascular disease (CVD). We aimed to compare the prevalence of glucose and lipid metabolism abnormalities, body fat amount and distribution, and the effect of body fat on glucose and lipid metabolism in Chinese and US teenagers.

**Methods:**

We included 5424 Chinese teenagers (48.5% male) from the China Child and Adolescent Cardiovascular Health (CCACH) study and 8704 USA teenagers (55.6% male) from the USA National Health and Nutrition Examination Survey (NHANES). Blood lipid, blood glucose, and body fat indicators were obtained using the same standardised measurements.

**Results:**

The prevalence of dyslipidaemia in Chinese teenagers was of those in the USA (hypercholesterolaemia = 3.5% vs 7.4%; high low-density lipoprotein cholesterol (LDL-C) = 3.6% vs 5.0%; low high-density lipoprotein cholesterol (HDL-C) = 9.9% vs 14.3%; hypertriglyceridaemia = 3.7% vs 10.1%) (*P* < 0.05). However, with the increase in body mass index (BMI), the prevalence of high LDL-C increased more in Chinese than in US teenagers, even exceeding them in the obese group (2.7% in non-overweight to 9.7% in overweight group in China, *P* < 0.05; 3.5% in non-overweight to 6.5% in the obese group in the USA, *P* < 0.05). The prevalence of impaired fasting glucose was higher in China than in the USA (28.0% vs 17.5%, *P* < 0.05). Besides, Chinese adolescents are more likely to accumulate fat in the abdomen, and the per-unit fat increase would bring a higher risk of dyslipidaemia in Chinese boys than in USA boys.

**Conclusions:**

Dyslipidaemia was more prevalent in US teenagers than in Chinese teenagers, but with the increase in BMI, the prevalence of high LDL-C increased more in Chinese than in US teenagers. Impaired fasting glucose (IFG) was significantly more prevalent in China than in the USA. The unfavoured body fat and higher risk of body fat on metabolic disorders in Chinese teenagers suggest that Chinese teenagers should pay more attention to the adverse effect of body fat on metabolic abnormalities.

Cardiovascular disease (CVD) remains the leading cause of death and disease burden in China and the USA [1,2]. As well-established CVD risk factors, glucose and lipid metabolism abnormalities showed different trends in the two countries. With the obesity epidemic, the prevalence of dyslipidaemia and diabetes in Chinese adults has increased by approximately 15% and 3% over the past decade, respectively [3-5], while remaining relatively stable in US adults [6,7]. Studies have identified these risk factors in childhood [8-11], during which the pathophysiological process of CVDs also begins [10,11]. However, few studies examined prevalence of glucose and lipid metabolism abnormalities in children and teenagers in both countries, and none directly compared the prevalence of glucose and lipid metabolism abnormalities in the two groups. Understanding and comparing the current status of glucose and lipid metabolism abnormalities between Chinese and USA children and teenagers can facilitate the development of effective strategies for preventing future CVD events.

Abnormalities in glucose and lipid metabolism are amplified in the presence of childhood obesity [12]. China has the largest number of children with obesity, and the US has the highest prevalence of childhood obesity in the world [3,13]. Both countries must explore glucose and lipid metabolism abnormalities from the perspective of obesity. Body mass index (BMI) is commonly used to assess obesity; however, it cannot differentiate between body fat and fat-free mass and cannot evaluate regional fat. Previous studies have found that the Chinese have a higher body fat percentage compared to US citizens in the same BMI category, and that body fat is more likely to accumulate in the abdomen region [14]. These differences in the amount and distribution of body fat may contribute to the differences in glucose and lipid metabolism between the two countries with the same BMI status [14,15]. However, these studies were all conducted in adults, and no paediatric studies compared the differences in the amount and distribution of body fat and the effect of body fat on glucose and lipid metabolism abnormalities between the Chinese and the USA.

We aimed to compare the prevalence of glucose and lipid metabolism abnormalities, body fat, and the effect of body fat on glucose and lipid metabolism abnormalities between Chinese and USA teenagers using data from two large-scale population-based cross-sectional studies, the China Child and Teenager Cardiovascular Health (CCACH) study and the USA National Health and Nutrition Examination Surveys (NHANES).

## METHODS

### Study population

Our study comprised two subsamples from the CCACH study and the NHANES in 1999-2018. The CCACH study was a large-scale cross-sectional survey conducted in China between 2013 and 2015, designed to evaluate cardiovascular health among Chinese urban children and adolescents aged 3-18 years [16]. First, we stratified the country into northern and southern regions by the Qinling-Huaihe line according to climate, economic development, and residents’ life habit. We selected Beijing, Changchun, Tianjin, Jinan, and Yinchuan from the northern region and Shanghai and Chongqing from the southern region. Next, we randomly selected several schools from each city according to the list of names obtained from the local education commissions. All students (n = 15 548) from the selected schools were invited to participate in the survey. The Ethics Committee of the Capital Institute of Pediatrics approved the study and we obtained written informed consent from all the participants and/or their parents.

NHANES is a nationally representative cross-sectional survey in the USA designed to evaluate adults’ and children's health and nutritional status. It is a continuous survey (1999-2023) relying on a stratified multistage probability sample based on selecting counties, blocks, households, and persons within households to represent the civilian, non-institutionalized US population [17]. The survey data and procedures are available elsewhere [18]. We combined data from 10 cycles (1999-2018) of NHANES and 101 316 participants in the original cross-sectional survey. The USA Centers for Disease Control and Prevention/National Center for Health Statistics Ethics Review Board approved NHANES. Written informed consent was obtained from all the participants and/or their parents.

**Figure 1:**
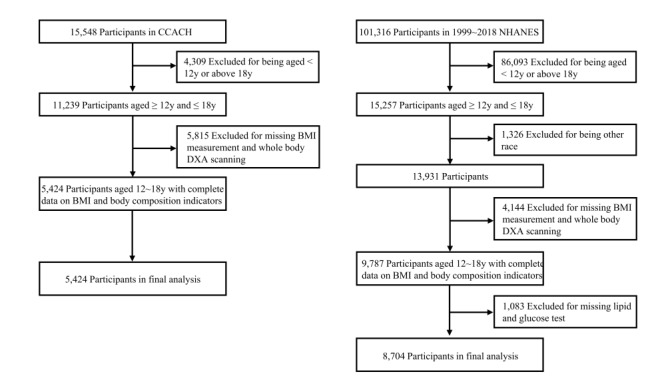
Flowchart of the sample selection from China (CCACH, 2013-2015) and the USA (NHANES, 1999-2018). CCACH – the China Child and Teenager Cardiovascular Health study, NHANES – the United States National Health and Nutrition Examination Survey, DXA – dual-energy x-ray absorptiometry.

In NHANES, only participants >12 years old were tested for fasting blood glucose. Therefore, this study selected teenagers aged 12-18 years from both CCACH (n = 11 239) and NHANES (n = 15 257) to maintain the uniformity of the age spectrum between China and the USA. We further excluded 5815 Chinese teenagers without anthropometric measurement or whole-body dual-energy x-ray absorptiometry (DXA) scanning. We excluded US teenagers whose ethnicity could not be determined (n = 1326), who were missing anthropometric measurement and whole-body DXA scanning (n = 4144), and who had no blood tests (n = 1083). Finally, 5424 Chinese and 8704 US teenagers were included in the analysis (**Figure 1**).

### Anthropometric measurements and whole-body DXA scanning

Both studies measured height and weight to the nearest 0.1 cm and 0.1 kg using standard methods and unified calibrated equipment. Subjects were asked to stand upright and wear lightweight clothing without shoes while taking measurements. Height and body weight were measured twice, and BMI was calculated as the mean weight in kilograms divided by the mean height in square meters. The age and sex-specific International Obesity Task Force (IOTF) criteria were used to define weight status as non-overweight, overweight, obesity, and severe obesity [19].

Whole-body DXA scanning was performed to test body fat indicators in both studies using Hologic fan-beam densitometers (Hologic, Bedford, Massachusetts, USA) by trained technicians according to the International Society of Clinical Densitometry’s (ISCD) standard operating procedure [20,21]. Fat mass percentage (FMP) was calculated as the proportion of a body’s fat mass (FM) concerning total body mass. Fat mass index (FMI, fat mass/height^2^) was calculated to account for the potential influence of body size or height. Fat-to-muscle mass ratio (FMR) was calculated as whole-body FM by whole-body muscle mass. The android-to-gynoid ratio (AGR) was calculated as the android region FM divided by the gynoid region FM, which is a proxy for body fat distribution. The android region is approximately the abdomen area from a line joining the two superior iliac crests and extended cranially for 20% of the distance between the base of the skull. The gynoid region lies the portion of the legs from the greater femoral trochanter, extending caudally to mid-thigh.

### Laboratory measurements

CCACH and NHANES collected blood specimens of participants after fasting for at least eight hours. In the two studies, total cholesterol (TC), triglyceride (TG), and fasting glucose were measured using the enzymatic method and high-density lipoprotein cholesterol (HDL-C) using the dimethod. LDL-C was calculated from measured values of TC, TG, and high-density lipoprotein (HDL) according to the Friedewald calculation: LDL = TC-HDL-(TG/5) [22,23]. Dyslipidaemia was defined as hypercholesterolaemia (TC≥5.18 mmol/L), high LDL-C (LDL-C≥3.37 mmol/L), low HDL-C (HDL-C<1.03 mmol/L), or hypertriglyceridemia (TG≥1.7 mmol/L) [24]. Impaired fasting glucose (IFG) was defined by the cut-point proposed by the American Diabetes Association guideline (≥5.6 mmol/L) [25].

### Self-reported questionnaire surveys

Demographics, lifestyle, and household income of the two studies were collected using self-reported questionnaires. Individuals from both studies were stratified into two groups by their frequency of dairy product consumption (daily vs nondaily). Physically active was defined as moderate to vigorous physical activity for more than one hour daily. Smoking status was categorized into non-smoker and smoker (current or previous smoking attempts). Poverty-income ratio (PIR) was used to reflect economic status, derived by dividing annual household income by the established city's poverty level, accounting for household size and year of assessment [26].

### Statistical analysis

We expressed continuous variables, such as age and PIR, as means and standard deviations (SDs), and categorical variables, such as age group, BMI category, and lifestyle proportion, as frequencies and percentages. We compared boys and girls or China and the USA using *t*-tests for continuous and χ^2^ tests for categorical variables.

We presented levels of body fat indicators (FMI, FMP, AGR, and FMR) in different BMI statuses as means and 95% confidence intervals (CIs) from the analysis of covariance (ANCOVA) adjusted for age. We calculated the crude prevalence and age-standardized prevalence rate (ASPR) with the 95% CIs of lipid and glucose metabolism abnormalities. We calculated ASPR using the combined population of CCACH and NHANES as the standard population. We calculated direct ASPR as follows [27]:



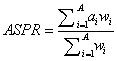



where w_i_ is the number of population in the *i*^th^ age group of the standard population and a_i_ is the age-specific rate in the *i*^th^ age group.

We performed multivariate logistical regression models with body fat indicators as the independent variable and glucose and lipid metabolism abnormalities as the dependent variable. The covariates included age, race, survey year, physical activity, daily milk consumption, smoking, PIR, and BMI. We estimated odds ratios (ORs) and 95% CIs for each unit increase in body fat indicators. We examined the differences in the effect between the two countries by adding the interaction term of specific body fat indicators with country into the models. We performed all statistical analyses using R software (version 4.1.2). We set the statistical value at *P* < 0.05 (two-sided).

## RESULTS

### General characteristics of the participants

This study included 5424 Chinese teenagers (2632 male, 48.5%, mean age = 15.1) and 8704 USA teenagers (4842 male, 55.6%, mean age = 15.0). The prevalence of overweight (15.9%), obesity (5.2%), and severe obesity (0.7%) in Chinese was significantly lower than in USA teenagers (23.0%, 12.1%, and 5.6%, respectively) (*P* < 0.05). Teenagers in China were less likely to exercise, have dairy daily, and smoke than those in the USA (all *P* < 0.05). Differences in BMI and lifestyles between the sexes were observed in both Chinese and USA teenagers. Chinese boys had a higher proportion of being overweight and obese and physically active than Chinese girls, while USA girls had a greater proportion of being overweight and obese and physically active than USA boys (*P* < 0.05). Boys in both China and the USA were more likely to be smokers than girls (**Table 1**).

**Table 1:** Characteristics of study population in CCACH and NHANES

	CCACH			NHANES		
**Characteristics**	**Male, n (%)**	**Female, n (%)**	**Total, n (%)**	**Male, n (%)**	**Female, n (%)**	**Total, n (%)**
Number of participants	2632 (48.5)	2792 (51.5)	5424 (100.0)	4842 (55.6)†	3862 (44.4)†	8704 (100.0)
Age in years, mean (SD)	15.0 (1.8)	15.2 (1.8)*	15.1 (1.8)	15.0 (2.0)	15.0 (2.0)	15.0 (2.0)
12-14 in years	993 (37.7)	910 (32.6)*	1903 (35.1)	2112 (43.6)†	1664 (43.1)†	3776 (43.4)†
15-18 in years	1639 (62.3)	1882 (67.4)*	3521 (64.9)	2730 (56.4)†	2198 (56.9)†	4928 (56.6)†
BMI category						
*Non-ow*	1881 (71.5)	2358 (84.5)*	4239 (78.2)	2936 (60.6)†	2225 (57.6)*†	5161 (59.3)†
*Ow*	505 (19.2)	359 (12.9)*	864 (15.9)	1110 (22.9)†	893 (23.1)†	2002 (23.0)†
*Ob*	215 (8.2)	68 (2.4)*	283 (5.2)	561 (11.6)†	492 (12.7)*†	1053 (12.1)†
*Severe ob*	31 (1.2)	7 (0.3)*	38 (0.7)	235 (4.9)†	253 (6.6)*†	488 (5.6)†
Physically active	297 (20.3)	233 (16.8)*	530 (18.6)	1004 (21.2)	1282 (33.8)*†	2286 (26.8)†
Daily dairy intake	222 (12.2)	202 (10.9)	424 (11.5)	1537 (35.3)†	1573 (44.6)*†	3110 (39.4)†
Smoker	504 (25.1)	189 (8.9)*	693 (16.8)	1613 (36.2)†	1088 (30.8)*†	2701 (33.8)†
PIR, mean (SD)	3.3 (2.6)	3.2 (2.7)	3.3 (2.7)	2.1 (1.5)†	2.0 (1.5)†	2.1 (1.5)†

BMI – body mass index, PIR – poverty-income ratio, Non-OW – non-overweight, OW – overweight, OB – obesity

*Differences in variables between sexes, *P* < 0.05.

†Differences in variables between China and the USA, *P* < 0.05.

### Prevalence of glucose and lipid metabolism abnormalities and levels of body fat in Chinese and USA teenagers

**Table 2** shows the crude prevalence and ASPRs of glucose and lipid metabolism abnormalities in Chinese and USA teenagers. IFG was the most prevalent glucose and lipid metabolism abnormality in both countries, affecting 28.0% of teenagers in China and 17.5% in the USA. Among the different forms of dyslipidaemia, the prevalence of low HDL-C was the highest in both countries, affecting 9.9% of Chinese teenagers and 14.3% of US teenagers. The trend of ASPRs across the BMI categories in both countries was similar: the prevalence of glucose and lipid metabolism abnormalities increased from non-overweight to overweight and obesity, except for LDL-C in the USA, which decreased from 7.9% in overweight to 6.5% in obesity. Compared with the USA, the ASPRs of dyslipidaemia in China were lower (hypercholesterolaemia = 3.5% vs 7.4%; high LDL-C = 3.6% vs 5.0%; low HDL-C = 9.9% vs 14.3%; hypertriglyceridemia = 3.7% vs 10.1%; all *P* < 0.05). However, with the increase in BMI, the prevalence of high LDL-C and low HDL-C increased more in Chinese than in USA teenagers, even exceeding them in the obese group. In contrast, the ASPRs of IFG in China were substantially higher than in the USA in all BMI categories (non-overweight = 25.5% vs 15.0%, overweight = 35.4% vs 17.2%, obesity = 38.2% vs 26.3%) (*P* < 0.05).

**Table 2:** The ASPRs and crude prevalence of glucose and lipid metabolism abnormalities in China and the USA % (95% CI)

	CN				USA			
	**Non-ow (n = 4239)**	**Ow (n = 864)**	**Ob (n = 321)**	**Total (n = 5424)**	**Non-ow (n = 5161)**	**Ow (n = 2002)**	**Ob (n = 1531)**	**Total (n = 8704)**
**Hyperchoesterolemia**								
ASPR	3.0 (2.9-3.1)	4.7 (4.5-4.9)	6.0 (5.4-6.6)	3.5 (3.4-3.5)	5.6 (5.5-5.6)*	8.9 (8.8-9.0)*	11.3 (11.3-11.4)*	7.4 (7.3-7.4)*
Crude prevalence	3.1 (3.0-3.2)	5.0 (4.7-5.3)	6.8 (6.1-7.5)	3.6 (3.5-3.6)	5.7 (5.6-5.7)	9.0 (8.9-9.1)	11.3 (11.2-11.4)	7.4 (7.3-7.4)
**High LDL-C**								
ASPR	2.7 (2.7-2.8)	5.4 (5.2-5.6)	9.7 (9.1-10.3)	3.6 (3.5-3.6)	3.5 (3.5-3.6)*	7.9 (7.7-8.1)*	6.5 (6.5-6.8)*	5.0 (5.0-5.1)*
Crude prevalence	2.8 (2.7-2.9)	5.4 (5.2-5.6)	10.2 (9.5-10.9)	3.7 (3.6-3.7)	3.5 (3.4-3.6)	8.1 (7.9-8.3)	6.6 (6.3-6.9)	5.1 (5.0-5.2)
**Low HDL-C**								
ASPR	7.1 (7.1-7.2)	17.1 (16.8-17.4)	31.7 (31.3-46.0)	9.9 (9.8-9.9)	8.6 (8.6-8.7)*	16.3 (16.2-16.4)	30.7 (30.7-30.8)	14.3 (14.3-14.3)*
Crude prevalence	7.0 (6.9-7.1)	16.1 (15.8-16.3)	28.8 (28.1-29.5)	9.7 (9.6-9.7)	8.5 (8.4-8.5)	16.5 (16.4-16.6)	30.6 (30.5-30.7)	14.3 (14.3-14.3)
**Hypertrigliceridemia**								
ASPR	2.1 (2.1-2.2)	7.1 (6.8-7.3)	17.0 (16.3-17.7)	3.7 (3.6-3.8)	5.5 (5.5-5.6)*	12.8 (12.7-12.9)*	22.2 (22.2-22.4)*	10.1 (10.1-10.2)*
Crude prevalence	2.1 (2.0-2.2)	7.0 (6.7-7.2)	15.6 (14.9-16.3)	3.7 (3.6-3.7)	5.6 (5.5-5.6)	12.9 (12.8-13.0)	22.3 (22.2-22.4)	10.2 (10.1-10.2)
**IFG**								
ASPR	25.5 (25.5-25.6)	35.4 (35.1-35.6)	38.2 (37.6-38.8)	28.0 (27.9-28.0)	15.0 (14.9-15.1)*	17.2 (17.0-17.4)*	26.3 (26.3-26.6)*	17.5 (17.4-17.5)*
Crude prevalence	24.8 (24.7-24.9)	35.5 (35.3-35.7)	40.0 (39.3-40.7)	27.4 (27.4-27.4)	15.1 (15.0-15.2)	17.4 (17.2-17.6)	26.3 (26.0-26.6)	17.6 (17.5-17.7)

CN – China, ASPR – age-standardized prevalence rate, LDL-C – low-density lipoprotein cholesterol, HDL-C – high-density lipoprotein cholesterol, IFG – impaired fasting glucose, CI – confidence interval, Non-OW – non-overweight, OW – overweight, OB – obesity

*Differences in ASPR between China and the US under the same BMI categories, *P* < 0.05.

**Figure 2:**
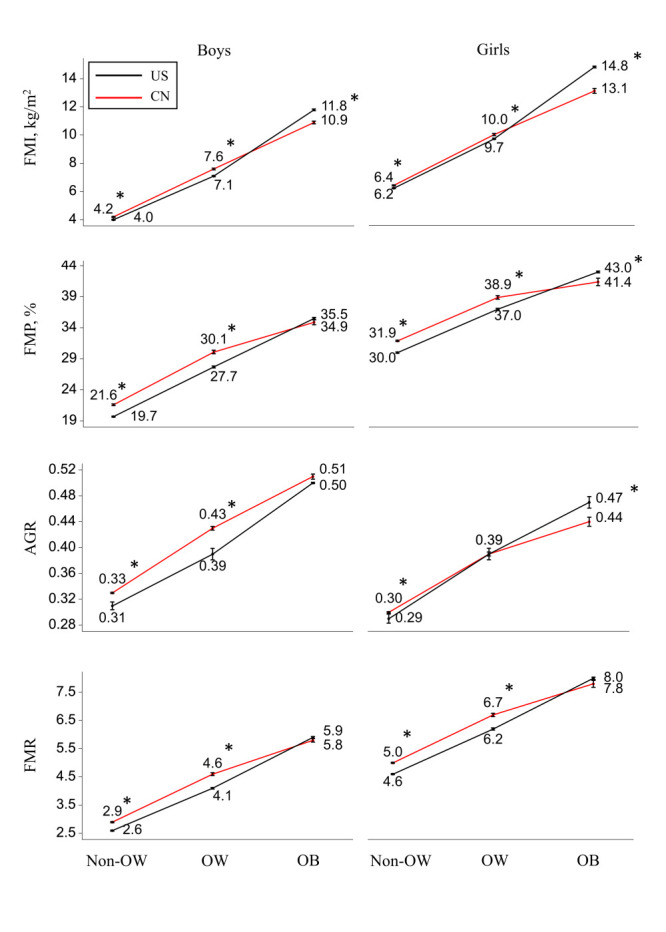
Body fat levels of Chinese and USA teenagers by BMI categories; Body fat levels were adjusted for age from ANCOVA models. *Differences in body fat levels between China and USA under the same BMI categories, *P* < 0.05. CN – China, Non-OW – non-overweight, OW – overweight, OB – obesity, FMI – fat mass index, FMP – fat mass percentage, AGR – android-to-gynoid fat ratio, FMR – fat-to-muscle ratio.

**Figure 2** shows the mean levels of body fat indicators (FMI, FMP, AGR, and FMR) in Chinese and USA teenagers by weight status. Both groups had the same pattern: body fat amount or distribution indicators increased dramatically from non-overweight to overweight and obesity. However, body fat amount indicators (FMI and FMP) were significantly higher in China for non-obese boys and girls (*P* < 0.05) and significantly higher in USA for obese boys and girls (*P* < 0.05). Notably, AGR was higher in Chinese than in USA boys in all BMI categories (0.33 vs 0.31 in non-overweight, 4.6 vs 4.1 in overweight, and 5.9 vs 5.8 in obesity).

### Association of body fat with glucose and lipid metabolism abnormalities in Chinese and USA teenagers.

**Table 3** presents the results of logistic regression analyses for body fat indicators (FMI, FMP, AGR, and FMR) with specific glucose and lipid metabolism abnormalities adjusted for covariates. AGR was associated with greater odds for hypercholesterolaemia, high LDL-C, hypertriglyceridaemia, and low HDL-C in both countries. Compared to body fat amount indicators (FMI and FMP), AGR showed a higher hazard of lipid metabolism, especially in Chinese boys (hypercholesterolaemia: OR = 5.24, 95% CI = 1.53-21.10; high LDL-C: OR = 2.90, 95% CI = 1.25-7.13; low HDL-C: OR = 2.18, 95% CI = 1.35-3.59; hypertriglyceridaemia: OR = 2.56, 95% CI = 1.04-6.46). In boys, FMI and FMP were associated with greater odds for all glucose and lipid metabolism abnormalities in both countries. However, compared with the USA boys, the per kg/m^2^ increase of FMI in Chinese boys had a more significant hazard for dyslipidaemia except for HDL-C, and the percentage increase of FMP in Chinese boys had a more significant hazard for high LDL-C and hypertriglyceridemia. In girls, FMI and FMP were associated with the risk of hypertriglyceridaemia in both countries (OR = 1.11, 95% CI = 1.00-1.22 in China; OR = 1.05, 95% CI = 1.01-1.08 in the US). However, FMI was only associated with the risk of Chinese girls’ hypercholesterolaemia and IFG. Additionally, per kg/m^2^ increase of FMI in Chinese girls had greater odds for hypertriglyceridaemia and hypercholesterolaemia than in US girls (hypertriglyceridaemia: OR = 1.25 vs 1.05; hypercholesterolaemia: OR 1.53 vs 1.13).

**Table 3:** Glucose and lipid metabolism abnormalities associated with 1-unit increase in body fat indicators in China and the US; Covariates in all models included age, race, survey year, physical activity, daily milk consumption, smoking, PIR, and BMI

	Boys					Girls				
	**CN**		**US**			**CN**		**US**		
	**OR (95% CI)**	***P*-value**	**OR (95% CI)**	***P*-value**	***P*-value for difference**	**OR (95%CI)**	***P*-value**	**OR (95% CI)**	***P*-value**	***P*-value for difference**
**Hypercholesterolaemia**										
FMI in kg/m^2^	1.73 (1.63-2.62)	0.003	1.21 (1.09-1.35)	<0.001	0.001	1.25 (1.02-1.53)	0.029	1.05 (0.91-1.20)	0.523	0.002
FMP in %	1.11 (1.04-1.21)	0.004	1.08 (1.05-1.11)	<0.001	0.207	1.06 (0.98-1.14)	0.152	1.03 (1.00-1.06)	0.086	0.080
AGR	5.24(1.53-21.10)	0.011	1.58 (1.27-1.97)	<0.001	0.009	1.81 (1.04-3.14)	0.035	1.28 (1.05-1.55)	0.013	0.076
FMR	1.08 (1.02-1.15)	0.008	1.03 (1.02-1.04)	<0.001	0.001	1.00 (0.95-1.06)	0.878	1.01 (1.00-1.02)	0.215	0.091
**High LDL-C**										
FMI in kg/m^2^	1.68 (1.25-2.32)	0.001	0.99 (0.83-1.18)	0.948	<0.001	1.19 (0.69-1.96)	0.520	1.12 (0.86-1.46)	0.394	0.220
FMP in %	1.11 (1.05-1.19)	0.001	1.05 (1.00-1.09)	0.041	0.022	1.07 (0.98-1.17)	0.123	1.06 (1.00-1.12)	0.061	0.815
AGR	2.90 (1.25-7.13)	0.016	1.62 (1.11-2.36)	0.012	0.022	2.22 (1.14-4.42)	0.020	2.01 (1.40-2.92)	<0.001	0.494
FMR	1.08 (1.03-1.13)	0.003	1.01 (1.00-1.03)	0.028	<0.001	1.04 (0.99-1.09)	0.124	1.02 (0.99-1.04)	0.142	0.535
**Low HDL-C**										
FMI in kg/m^2^	1.26 (1.07-1.49)	0.006	1.09 (1.02-1.18)	0.013	0.471	0.73 (0.51-1.04)	0.078	1.10 (0.98-1.23)	0.111	0.362
FMP in %	1.05 (1.00-1.10)	0.049	1.04 (1.02-1.06)	<0.001	0.574	1.01 (0.97-1.04)	0.715	1.04 (1.01-1.07)	0.005	0.496
AGR	2.18 (1.35-3.59)	0.002	1.82 (1.55-2.13)	<0.001	0.071	1.38 (1.06-1.78)	0.015	1.61 (1.34-1.92)	<0.001	0.356
FMR	1.04 (1.01-1.08)	0.010	1.01 (1.01-1.02)	0.001	0.946	0.99 (0.98-1.01)	0.460	1.01 (1.00-1.02)	0.023	0.559
**Hypertriglyceridaemia**										
FMI in kg/m^2^	1.92 (1.33-3.12)	0.002	1.25 (1.14-1.36)	<0.001	<0.001	1.53 (1.23-1.90)	<0.001	1.13 (1.09-1.16)	<0.001	0.046
FMP in %	1.13 (1.05-1.25)	0.003	1.10 (1.07-1.12)	<0.001	0.082	1.11 (1.00-1.22)	0.047	1.05 (1.01-1.08)	0.007	0.903
AGR	2.56 (1.04-6.46)	0.041	2.03 (1.71-2.41)	<0.001	0.005	4.09 (1.76-8.48)	0.002	2.13 (1.78-2.56)	<0.001	0.414
FMR	1.11 (1.04-1.18)	0.001	1.04 (1.03-1.05)	<0.001	0.001	1.07 (1.02-1.12)	0.006	1.01 (1.00-1.03)	0.020	0.392
**IFG**										
FMI in kg/m^2^	1.14 (1.07-1.21)	<0.001	1.14 (1.05-1.23)	0.002	0.338	1.12 (1.03-1.23)	0.007	1.08 (0.92-1.25)	0.348	0.324
FMP in %	1.03 (1.01-1.04)	<0.001	1.04 (1.02-1.06)	<0.001	0.275	1.03 (1.01-1.05)	0.005	1.01 (0.98-1.05)	0.558	0.430
AGR	0.90 (0.60-1.35)	0.618	1.44 (1.22-1.69)	<0.001	<0.001	0.94 (0.79-1.11)	0.463	1.10 (0.89-1.34)	0.370	0.868
FMR	1.02 (1.01-1.03)	<0.001	1.02 (1.01-1.03)	0.003	0.771	1.01 (1.00-1.02)	0.016	1.00 (0.99-1.02)	0.548	0.429

CN – China, FMI – fat mass index, FMP – fat mass percentage, AGR – android-to-gynoid fat ratio, FMR – fat-to-muscle ratio, LDL-C – low-density lipoprotein cholesterol, HDL-C – high-density lipoprotein cholesterol, IFG – impaired fasting glucose, OR – odds ratio, CI – confidence interval

## DISCUSSION

The CCACH and NHANES cross-sectional studies provided a unique opportunity for this comparative study. We primarily found that the prevalence of dyslipidaemia was lower in China than in the USA, but with the increase in BMI, the prevalence of high LDL-C and low HDL-C increased more in Chinese than in US teenagers, even exceeding them in the obese group. In contrast, the prevalence of IFG was higher in China than in the USA. Furthermore, we found that Chinese teenagers had higher body fat mass and FMP than USA teenagers, especially in those of non-obese status, and body fat in Chinese boys was more likely to accumulate in the abdominal region. We examined the associations between body fat and glucose and lipid metabolism abnormalities and found that the per-unit increase of body fat in Chinese boys would bring a higher risk of dyslipidaemia except for low HDL-C compared to USA boys.

Although dyslipidaemia has been considered challenging mostly in developed countries, its prevalence is increasing in China due to BMI status changes. The nationwide survey showed that the prevalence of dyslipidaemia in Chinese adults increased from 18.6% in 2004 to 33.8% in 2019 [4,5], while in the USA, the prevalence of dyslipidaemia remained relatively constant at 23% from 2003-2004 to 2013-2014 [6]. Our results extended these findings to the teenager of the two countries: the prevalence of hypercholesterolaemia, high LDL-C, HDL-C, and hypertriglyceridaemia were all lower in Chinese than in US teenagers, but the prevalence of high LDL-C and low HDL-C in China ascends more obviously with the increase of BMI. Studies indicated that glucose and lipid metabolism abnormalities during childhood might predict the early onset of CVD morbidity and mortality in adulthood [10,28], and these CVD consequences may be reversible when a child normalizes his or her BMI before adulthood [29,30]. These findings suggest that Chinese teenagers need to be more attentive of the increase of dyslipidaemia caused by BMI changes and highlight the crucial importance of weight control and obesity prevention in teenagers in counteracting CVDs.

Although BMI is strongly associated with CVD risks, it is limited by not discriminating against body composition and ignoring body fat distribution. Recently, DXA was developed as a precise method to quantify body fat and identify body fat distribution. A survey of 1349 children aged 6-18 years in Austria showed that a higher DXA-measured FMI was associated with higher triglycerides and LDL [31]. We found a similar pattern in Chinese and US boys: DXA-measured body fat amount indicators (FMI and FMP) were positively associated with dyslipidaemia. Furthermore, we used AGR as a proxy for body fat distribution and found that body fat in Chinese teenagers tended to accumulate in the abdomen, especially in Chinese boys. A previous study reported that AGR was a much stronger independent predictor of cardiometabolic variables than FMP [32]. We similarly found that AGR had a more significant effect on lipid metabolism than body fat amount indicators, i.e. FMI and FMP, possibly because individuals with high AGR probably have higher visceral adiposity, which secretes more proinflammatory cytokines [33,34]. Additionally, we found that a per-unit increase of AGR in Chinese boys would bring a higher risk of dyslipidaemia compared to USA boys. These findings indicate that Chinese boys should be more attentive of the adverse effect of abdominal adiposity on dyslipidaemia than their US counterparts.

Furthermore, it should be noted that the prevalence of IFG was significantly higher in Chinese than in US teenagers in all BMI categories. Previous studies showed that the IFG prevalence in China was higher than in European countries. A study of 3978 Danish children found that the prevalence of IFG with normal weight, overweight, and obesity were 4.1%, 9.9%, and 14%, respectively [35]. Another study of 35 633 European children aged 2-18 years showed that the prevalence of IFG was only 5.7% in Germany and 17.1% in Sweden [36]. The following hypotheses could explain the high prevalence of IFG in Chinese teenagers. First, the Chinese have more abdominal and visceral fat accumulation, which is more insulin resistant [33,37,38]. However, these findings were found in adults. We found the same result in Chinese and US teenagers: abdominal fat measured by DXA was deposited more in Chinese than in US boys in the same weight status. Second, the Chinese diet is characterized by refined cereals and low-dairy milk, which is associated with a higher risk of diabetes [39]. Third, we found that the proportion of physically active Chinese teenagers was 18.6%, significantly lower than that of USA teenagers. Studies have observed that physical inactivity was the independent risk factor for increased diabetes [40,41].

This study has several strengths. It is the first to compare the prevalence of glucose and lipid metabolism abnormalities and body fat on glucose and lipid metabolism abnormalities on data from two large samples from the USA and China. Second, we used ASPR to compare the prevalence of glucose and lipid metabolism abnormalities considering inter-population comparisons. Third, anthropometric, body fat, and blood indicators were obtained using standardized measurements in both CCACH and NHANES. However, this study also has some limitations. First, the cross-sectional design of the CCACH and NHANES makes it challenging to determine a causal relationship between body fat indicators and glucose and lipid metabolism abnormalities. Second, the specific study period was not identical between China (2013-2015) and the USA (1999-2018), but the median times for the data collection were similar.

## CONCLUSIONS

Dyslipidaemia was more prevalent in USA teenagers than in Chinese teenagers, but with the increase of BMI, the prevalence of high LDL-C and low HDL-C increased more in Chinese than in USA teenagers, even exceeding them in the obese group. IFG was more prevalent in Chinese than in USA teenagers. The unfavoured body fat and higher risk of body fat on metabolic disorders in China suggest that Chinese teenagers should be more attentive of the adverse effect of body fat, especially abdominal fat distribution on glucose and lipid metabolism abnormalities, than USA teenagers.
